# A National Responsibility

**Published:** 1920-08-21

**Authors:** 


### The Hospital

The Workers' Newspaper of Administrative Medicine and Institutional Life, Administration, National Insurance and Health.

No. 1785, Vol. LXVIII. SATURDAY, AUGUST 21, 1920. PRICE TWOPENCE

### A NATIONAL RESPONSIBILITY.

Dr. Addison's long-expected Health Bill was introduced by him into the House of Commons just before the adjournment on Monday last. It is described as a Bill "to amend the law relating to the Housing of the People, Public Health, and Local Government, and for purposes in connection therewith," and its twenty-six clauses are divided into two sections. The provisions of Part I. include power to hire dwelling-houses compulsorily for housing the working classes, to provide houses for employees of local authorities, and to amend the Housing (Additional Powers) Act, 1919, so as to give power to the Minister of Health to prohibit or restrict the construction of buildings in a certain area.

The most important clause of the Bill, however, is that relating to hospitals. Clause 11 of Part II. gives power to the Council of a County to supply and maintain hospitals (including out-patient departments) for the treatment of illness and disease generally or any particular class of illness or disease; to contribute on such terms and conditions as may be approved by the Minister to any voluntary hospitals or similar institutions within their area; and to undertake the maintenance of any Poor-Law hospitals or infirmaries within their area. Powers are given to the Councils to recover the expense of the maintenance in such a hospital of a person who is not a pauper, in the same way as a local authority has this power under the Public Health Act, 1875. Clause 11 also gives power to the Council of a County to establish and maintain or to contribute towards the cost of establishing or maintaining an ambulance service.

The principle of this clause seems to us almost wholly vicious and impracticable. It strikes quite definitely at the root of the voluntary principle; for private charity will certainly dry up if the voluntary hospitals are supported out of the rates. Yet it does nothing to substitute any coherent, well-considered, national line of policy for the institutional treatment of the sick. The problem is a national one, and any solution, to be satisfactory, must be based on national, not parochial, requirements. Hitherto we have scraped along in the usual illogical British fashion, with a combination of parochial (that is, Poor-Law) institutional provision and of the wider and much more elastic voluntary system, which has filled in the gaps of the rigid, inelastic, and purely local system. If the voluntary principle is to be abandoned, which is what this clause will bring about—whether intentionally or not—some properly organised national, not local, system must take its place. Is it conceivable for a moment that the ratepayers of London or of any other large city, full of hospitals taking patients from all over the country, are going to tax themselves for the benefit of the inhabitants of the neighbouring six or eight counties? Local authorities which contribute from the rates are certain to demand in return "most favoured" privileges for their inhabitants, a demand which the Ministry of Health will be bound to concede. The voluntary hospitals will become the sport of parochial politics, into whose vortex they will have to plunge as the price of continued existence. Their fate will be settled by chance aggregations of county councillors, profoundly ignorant for the most part of what hospitals do and of what they need, and elected to power on far other issues than the health of their constituents.

If it be objected to these forebodings that the Ministry of Health will see to all that, will supervise the local authorities and prevent them from wrecking the hospitals by their interference, the answer is, in the first place, that the Ministry of Health is a bureaucracy and that it appears to be asking for a blank cheque which it cannot safely be given; and in the second, that the local councils are not obliged to contribute, and that they have only to refuse to do so in order to bend the Ministry to their will. Give local authorities the power of the purse, and those authorities, in spite of every

Civil Servant in the land, will control hospital policy in their own areas. The more this clause is studied, the more hopelessly impossible it appears; dare the suggestion be hazarded that it is not intended seriously, but is merely a kite to test the wind of public opinion? Not by bringing the nation's health into the realm of parish-pump politics is the voluntary system to be uprooted: whatever views any of our readers may hold as to the question of State support *versus* voluntary effort, surely none of them can see good in this ill-devised half-and-half measure. The Ministry of Health must really try again, and show more evidence of breadth of view.

